# oxLDL antibody inhibits MCP‐1 release in monocytes/macrophages by regulating Ca^2+^/K^+^ channel flow

**DOI:** 10.1111/jcmm.13033

**Published:** 2016-12-20

**Authors:** Jinyu Su, Hui Zhou, Xianyan Liu, Jan Nilsson, Gunilla Nordin Fredrikson, Ming Zhao

**Affiliations:** ^1^ Department of PathophysiologyKey Lab for Shock and Microcirculation Research of GuangdongSouthern Medical UniversityGuangzhouChina; ^2^ Department of Clinical SciencesScania University HospitalMalmö Lund UniversityMalmöSweden

**Keywords:** oxLDL, MAPKs, MCP‐1, FcgammarRIIB, atherosclerosis, inward rectifier K^+^ channel, Ca^2+^, BI‐204

## Abstract

oxLDL peptide vaccine and its antibody adoptive transferring have shown a significantly preventive or therapeutic effect in atherosclerotic animal model. The molecular mechanism behind this is obscure. Here, we report that oxLDL induces MCP‐1 release in monocytes/macrophages through their TLR‐4 (Toll‐like receptor 4) and ERK MAPK pathway and is calcium/potassium channel‐dependent. Using blocking antibodies against CD36, TLR‐4, SR‐AI and LOX‐1, only TLR‐4 antibody was found to have an inhibitory effect and ERK MAPK‐specific inhibitor (PD98059) was found to have a dramatic inhibitory effect compared to inhibitors of other MAPK group members (p38 and JNK MAPKs) on oxLDL‐induced MCP‐1 release. The release of cytokines and chemokines needs influx of extracellular calcium and imbalance of efflux of potassium. Nifedipine, a voltage‐dependent calcium channel (VDCC) inhibitor, and glyburide, an ATP‐regulated potassium channel (K^+^_ATP_) inhibitor, inhibit oxLDL‐induced MCP‐1 release. Potassium efflux and influx counterbalance maintains the negative potential of macrophages to open calcium channels, and our results suggest that oxLDL actually induces the closing of potassium influx channel – inward rectifier channel (K_ir_) and ensuing the opening of calcium channel. ERK MAPK inhibitor PD98059 inhibits oxLDL‐induced Ca^2+^/K_ir_ channel alterations. The interfering of oxLDL‐induced MCP‐1 release by its monoclonal antibody is through its FcγRIIB (CD32). Using blocking antibodies against FcγRI (CD64), FcγRIIB (CD32) and FcγRIII (CD16), only CD32 blocking antibody was found to reverse the inhibitory effect of oxLDL antibody on oxLDL‐induced MCP‐1 release. Interestingly, oxLDL antibody specifically inhibits oxLDL‐induced ERK MAPK activation and ensuing Ca^2+^/K_ir_ channel alterations, and MCP‐1 release. Thus, we found a molecular mechanism of oxLDL antibody on inhibition of oxLDL‐induced ERK MAPK pathway and consequent MCP‐1 release.

## Introduction

Atherosclerosis is an inflammatory disease induced by imbalance of lipid metabolism – hyperlipaemia 1. Oxidation of low‐density lipoprotein (oxLDL) induced not only macrophage uptake and foam cell formation, but also T cell reaction and even antibody production by B cells 2, 3, 4. Cytokine expression in arteriosclerotic animal model serum has a Th1 immune profile. Atherosclerosis is now regarded as an autoimmune disease 5. Vaccination of oxLDL peptide or adoptive transfer of antibodies against oxLDL to arteriosclerotic animal model has exhibited preventive or therapeutic effects 6. Plaque area after immune therapies reduced to 50% 7. Moreover, macrophage staining and MCP‐1 release assay have shown that inflammation decreased after oxLDL antibody treatment. The molecular mechanisms of antibody regulation of inflammatory reaction are less obvious.

We previously reported that bone marrow cells from FcγRIIB‐deficient mice which were transplanted to low‐density lipoprotein receptor‐deficient (LDLR^−/−^) mice induced atherosclerotic lesion area in the descending aorta about fivefold larger than in LDLR^−/−^ control mice 8. Using mac‐1 and P‐ERK MAPK antibody staining of splenocytes, it was found that ERK activation in bone marrow‐transplanted mice was significantly higher than in control mice (unpublished results). These results give a clue that inhibition of inflammatory reaction by oxLDL antibody might not be through its neutralizing effect but through its FcγRIIB and ERK MAPK signal transduction pathway.

MAPKs were activated when monocytes/macrophages were exposed to oxLDL 9. At least three major groups of MAP kinases have been identified in mammalian cells so far: *(i)* extracellular signal‐regulated kinase (ERK), *(ii)* c‐Jun N‐terminal kinase (JNK) or stress‐activated protein kinase (SAPK) and *(iii)* p38 MAP kinase. The ERK pathway is preferentially activated by growth‐related stimuli, while JNK and p38 pathways are often linked with cellular stress 10. It is reported that oxLDL activates cellular signal transduction through its scavenger receptors. The major responsible scavenger receptors for oxLDL uptake and activation of monocytes/macrophages are SR‐AI (scavenger receptor AI), CD36 (cluster of differentiation 36), LOX‐1 (lectin‐like Ox‐LDL receptor 1) and TLR‐4 11, 12, 13, 14, 15.

Monocyte chemoattractant protein‐1 (MCP‐1/CCL2) is one of the key chemokines that regulates migration and infiltration of monocytes/macrophages into the lesion area. It is overexpressed in patients with atherosclerosis 16. MCP‐1 release involves Ca^2+^ activity inside the cell 17. The Ca^2+^ channel is voltage‐dependent, and the extent of Ca^2+^ influx depends on the degree of cell membrane potential polarization. The more the negative potential on the cell membrane, the more the Ca^2+^ influx into cytoplasm when the Ca^2+^ channel is activated 18, 19. The maintenance of cell membrane potential relies on the ratio of outward to inward K^+^ current. Thus, K^+^ outward current increase or K^+^ inward current decrease may result in cell membrane potential hyperpolarization 20, 21, 22, 23. Here, we report that oxLDL mAb inhibited monocyte MCP‐1 release and mRNA expression in a dose‐dependent manner in the antibody treatment experiment 24. We used *in vitro* study of oxLDL‐induced monocyte/macrophage MCP‐1 release model to investigate the molecular mechanism of oxLDL mAb on inhibition of MCP‐1 release and its cellular signal transduction pathways. We found that oxLDL mAb inhibits MCP‐1 release through its FcγRIIB, regulating oxLDL→TLR‐4→ERK MAPK→K_ir_ closure→Ca^2+^ channel opening‐mediated MCP‐1 release. The findings may reveal the molecular mechanism of how oxLDL mAb might be able to inhibit inflammatory reaction in atherosclerotic animal model.

## Materials and methods

### Materials

DMEM, foetal bovine serum (FBS), Dulbecco's phosphate‐buffered saline (DPBS) and HEPES were purchased from Invitrogen (Burlington, ON, Canada). Human MCP‐1 ELISA Kit was from Uscn Life Science Inc. (Houston, TX, USA). Nifedipine, glyburide and dimethyl sulfoxide (DMSO) were obtained from Sigma‐Aldrich (St. Louis, MO, USA). Fluo‐4‐AM and Pluronic F‐127 were purchased from DOJINDO (Rockville, MD, USA). Antibodies recognizing phosphorylation of P‐ERK, P‐JNK, P‐p38 and P‐c‐jun were from Cell Signaling Technology (Danvers, MA, USA). Antibodies recognizing LOX‐1, SR‐AI and CD36 were purchased from Abcam (Cambridge, MA, USA). Inhibitors of ERK (PD98059), JNK (SP600125) and p38 (SB203580) were from Beyotime (Beijing, China). Antibodies of CD16, CD32 and CD64 were purchased from Santa Cruz Biotechnology Inc. (Dallas, Texas, USA). The oxLDL monoclonal antibody (BI‐204) and control antibody (FITC‐8) were kindly provided by BioInvent International AB (Lund, Sweden). Antibodies recognizing TLR‐4 and β‐actin were from Proteintech Group Inc. (Chicago, IL, USA). BCA protein assay reagents, BSA standards and SuperSignal Femto substrate were purchased from Pierce (Milwaukee, WI, USA).

### Ethical statement

The study design was approved by Southern Medical University ethics board, and the performance was followed according to the Helsinki declaration. Written informed consent was obtained from all donators prior to treatment.

### Preparation of CD14^+^ human monocytes and RAW264.7 cells

Monocytes were prepared from human peripheral blood mononuclear cells (PBMCs), as described previously 25. Briefly, venous blood from healthy volunteers was heparinized and layered over Ficoll‐Isopaque (Pharmacia, Freiburg, Germany) density gradient reagent according to the manufacturer's instructions; mononuclear cells were separated by centrifugation at 400 × g for 30 min. at room temperature. Mononuclear cells were collected and washed two times with PBS without Ca^2+^ and Mg^2+^ by centrifugation at 250 × g for 20 min. at 4°C. Cells were then diluted with complete RPMI 1640 medium. Human monocytes were purified with MACS CD14 microbeads (Miltenyi Biotec GmbH, Bergisch Gladbach, Germany) according to the manufacturer's instructions.

Murine macrophage cell line RAW264.7 was from American Type Culture Collection (ATCC). It was grown in complete DMEM medium under standard tissue culture conditions.

### Preparation of oxLDL, lipid(‐) serum and oxLDL(‐) serum

Healthy human serum was from Nanfang Hospital. Lipid‐depleted serum and LDL (*d* = 1.020–1.063 g/ml) were isolated by sequential ultracentrifugation. LDL was incubated with 5 μmol/L CuSO_4_ (oxLDL) or 1 μmol/L FeSO_4_ (mmLDL) at 37°C for 24 hrs as described previously 26. LDL, oxLDL and mmLDL were sterilized and stored at 4°C in the dark and used within 2 weeks. The protein concentration was determined by the Bradford method 27. oxLDL and mmLDL were analysed by thiobarbituric acid‐reactive substances (TBARS) and electrophoretic mobility 28. oxLDL(‐) serum was prepared from oxLDL‐containing human serum through depletion of oxLDL by BI‐204‐conjugated column.

### Intracellular Ca^2+^ imaging

RAW264.7 cells were seeded in confocal dishes and grown for 24 hrs. Fluo‐4‐AM was dissolved in DMSO containing 20% Pluronic F‐127. Macrophages were incubated with 10 μM Fluo‐4‐AM in physiological salt solution (final concentration of DMSO was 0.1%) at room temperature for 30 min. Cells were then washed and incubated in HEPES‐buffered medium with nifedipine or inhibitors of ERK, JNK and p38, respectively, for 30 min. at room temperature. Medium was then removed and replaced with fresh media containing compounds and inhibitors as indicated. Fluorescence was measured every 0.6 sec. for 5 min. Image analysis was performed using Zeiss LCS software, and fluorescence of every cell in each field was measured. On average, 78.6 ± 12.4 cells were separately analysed per condition in each experiment. Cells exhibiting an increase in fluorescence of at least two times that of background, followed by a decrease in fluorescence and another increase in fluorescence, were scored as positive calcium oscillations. Each inhibitor was performed in duplicate within the experiment, and data shown are representative of at least three independent experiments.

### Electrophysiological recordings

The whole‐cell configuration of the patch clamp technique 29 was used to voltage clamp macrophages at room temperature. Patch pipettes pulled (P 97; Sutter Instruments, Novato, CA, USA) from borosilicate glass (Clark Electrochemical Instruments, Reading, UK) had resistances of 5–6 MΩ when filled with pipette solution. The seal resistances determined before rupturing the membrane patch within the tip of the pipette were over 1 GΩ. Compensation for capacitance and series resistance was achieved by the integrated circuitry of the patch amplifier. The bath (extracellular) solution for whole‐cell current measurement contained NaCl (135 mM), KCl (5 mM), MgCl_2_ (1 mM), CaCl_2_ (1.8 mM), HEPES (N‐[2‐hydroxyethyl]piperazine‐N′‐[2‐ethanesulphonic acid]) (10 mM) and glucose (10 mM); the pH was adjusted to 7.4 using 1 mM NaOH. The pipette solution contained KCl (140 mM), MgCl_2_ (2 mM), CaCl_2_ (1 mM), HEPES (10 mM) and EGTA (ethyleneglycol‐bis‐(b‐aminoethylether)‐N,N,N′,N′‐tetraacetic acid) (11 mM); the pH was adjusted to 7.4 with 1 mM KOH. The ratio of EGTA/CaCl_2_ in this solution set the free intracellular Ca^2+^ to ~ 10 nmol 30. To suppress K^+^ currents, KCl in the bath solution was replaced by CsCl (5 mM) and the pH was adjusted to 7.4 with 1 mM CsOH.

The patch clamp experiments were performed as described previously 31 using Micromanipulator MP‐285 and MultiClamp 700B patch clamp amplifier (Axon Instruments, Union City, CA, USA). Signals were low‐pass‐filtered at 5 kHz (low‐pass Bessel filter), digitized (sample rate: 10 kHz) using a Digidata 1440A converter (Axon Instruments) and stored and analysed using pClamp 10.2 software (Axon Instruments). The computer and software system was also used for generating voltage and current pulses. Voltage‐dependent currents were evoked by voltage pulses of 300‐msec. duration delivered every 5 sec. from a holding potential of −50 mV in 10‐mV increments. The steps ranged from −170 to +70 mV. Independent experiments were repeated, and 5–10 cells in each group were measured (*n* = 5).

### Western blotting

Cells were lysed with M‐PER Protein Extraction Reagent (Pierce, Rockford, IL, USA) supplemented with protease and phosphatase inhibitor cocktail, and protein concentrations of the extracts were measured by bicinchoninic acid (BCA) assay (Pierce). Forty micrograms of the protein was used and loaded per lane, subjected to sodium dodecyl sulphate–polyacrylamide gel electrophoresis (SDS‐PAGE), transferred onto nitrocellulose membranes and then blotted as described previously 32. Detection antibodies used (mentioned above in the section ‘Materials’) recognized phosphorylation of ERK, JNK, p38 and c‐jun. The β‐actin antibody was used as a control.

### MCP‐1 ELISA

The Human MCP‐1 ELISA Kit was employed to assay cell culture conditioned medium and carried out according to the manufacturer's instructions (Uscn Life Science Inc.). CD14^+^ monocytes were pre‐treated with indicated treatments and further incubated under standard tissue culture conditions for 2 days. The cytokine levels in the cell culture media were detected by a biotin‐labelled antibody and HRP‐conjugated streptavidin and measured at a wavelength of 450 ± 10 nm.

### Statistical analysis

The results were expressed as means ± S.D. One‐way anova and Student's *t*‐test from an SPSS software package were used for statistical evaluation. *P*‐values <0.05 were considered significant: *: *P* < 0.05; **: *P* < 0.01; ***: *P* < 0.001.

## Results

### oxLDL induces monocyte/macrophage MCP‐1 release through TLR‐4‐ and MAPK‐dependent pathways

**Figure 1:**
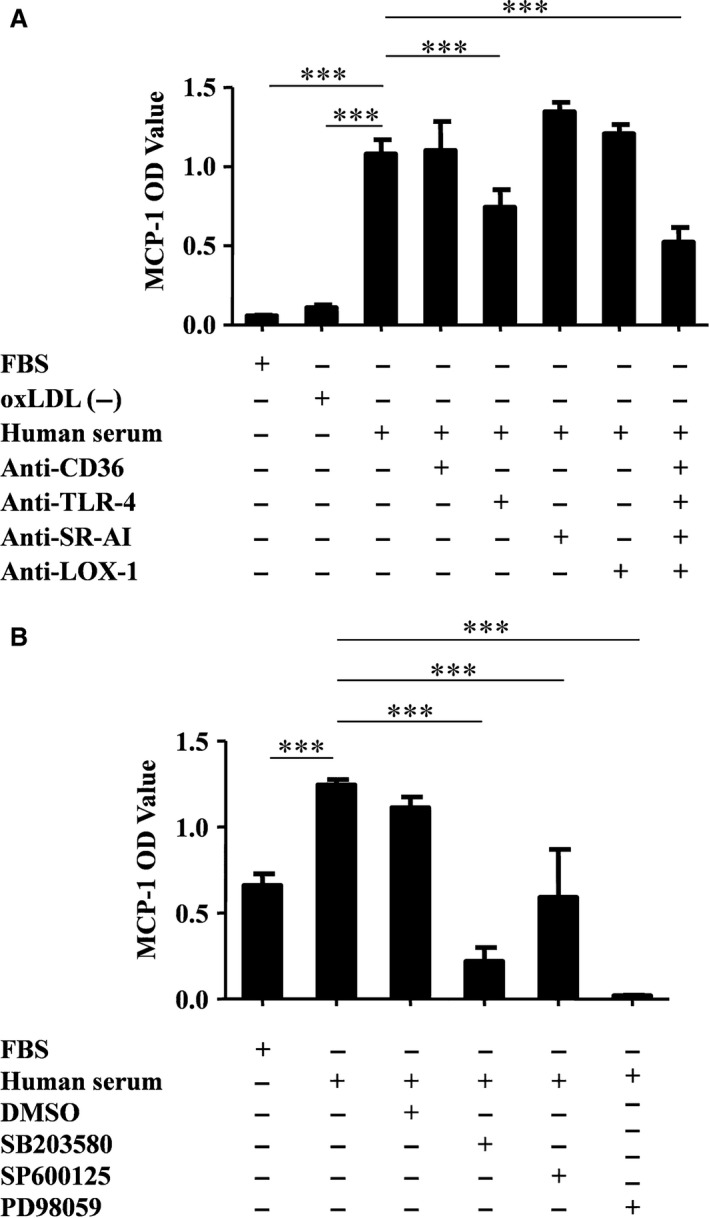
oxLDL induces monocyte/macrophage MCP‐1 release through TLR‐4‐ and ERK MAPK‐dependent pathways. Primary CD14^+^ monocytes were activated either by oxLDL‐containing human serum or foetal bovine serum (FBS) or by oxLDL antibody column‐treated control human serum (oxLDL(‐)). MCP‐1 release from CD14^+^ monocytes in culture medium was tested with ELISA. (**A)** Cells were pre‐treated with CD36, TLR‐4, SR‐AI and LOX‐1 blocking antibodies, respectively. ***P = 0.0001, one‐way anova. All data are shown as means ± S.D. (**B**) Cells were pre‐treated with inhibitors of JNK (SP600125), ERK (PD98059) and p38 (SB203580) MAPKs, respectively (*n* = 3). ***P = 0.0001, one‐way anova. All data are shown as means ± S.D.

oxLDL‐containing human serum (has been tested compare to FBS in Figure S2) and oxLDL (30 mg/ml) in the presence of 10% FBS both induced CD14^+^ monocyte/macrophage MCP‐1 release (Fig. 1A and Figure S5), whereas human serum treated in an oxLDL antibody‐conjugated agarose column, named oxLDL(‐) serum, had no effect (Fig. 1A). To test which receptor mediated the oxLDL‐induced MCP‐1 release, we explored blocking antibodies against CD36, TLR‐4, SR‐AI and LOX‐1. Only the TLR‐4 blocking antibody showed an inhibitory effect, and simultaneous administration of the four blocking antibodies had no synergistic effect (Fig. 1A and Figure S5). To further evaluate which MAPK might be involved in oxLDL‐containing human serum‐induced monocyte/macrophage MCP‐1 release, p38, ERK and JNK MAPK inhibitors were investigated. All these signal transduction pathways might be involved, but blocking the ERK MAPK pathway resulted in a more dominant inhibition (Fig. 1B).

### oxLDL induces monocyte/macrophage MCP‐1 release that is ATP‐regulated potassium/calcium channel‐dependent

**Figure 2:**
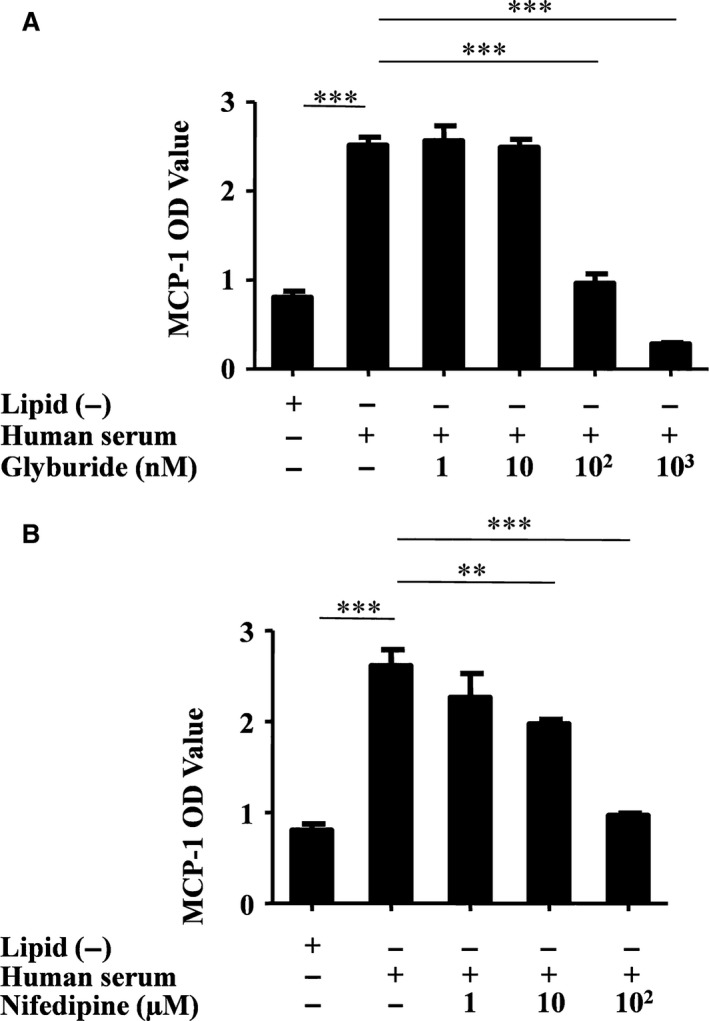
Human serum‐induced MCP‐1 release is Ca^2+^/K^+^ channel‐dependent. Primary CD14^+^ monocytes were activated either by oxLDL‐containing human serum or by lipid‐depleted serum (Lipid(‐)). MCP‐1 released from the CD14^+^ monocytes into the culture medium was tested with ELISA. (**A**) CD14^+^ monocytes were pre‐treated with the potassium channel inhibitor glyburide (1 nM, 10 nM, 100 nM, 1 μM respectively) and then exposed to oxLDL‐containing human serum or control lipid‐depleted serum. ***P = 0.0001, one‐way anova. All data are shown as means ± S.D. (**B**) CD14^+^ monocytes were pre‐treated with the calcium channel inhibitor nifedipine (1 μM, 10 μM and 100 μM, respectively) and then exposed to oxLDL‐containing human serum or control lipid‐depleted serum (*n* = 3). **P = 0.0035, ***P = 0.0001, one‐way anova. All data are shown as means ± S.D.

The release of cytokines and chemokines from monocytes/macrophages needs Ca^2+^ ion involvement, and Ca^2+^ and K^+^ ions have a charge balance relation in the cytosol 17, 33. Influence on the intracellular K^+^ concentration by its ATP‐regulated channel (K^+^_ATP_) inhibitor (glyburide; Fig. 2A) or the calcium concentration by the Ca^2+^ channel inhibitor (nifedipine; Fig. 2B) resulted in significantly reduced human serum‐induced MCP‐1 secretion from monocytes/macrophages in a dose‐dependent manner.

### oxLDL induces inward rectifier K^+^ (Kir) channel repression in RAW264.7 cells

**Figure 3:**
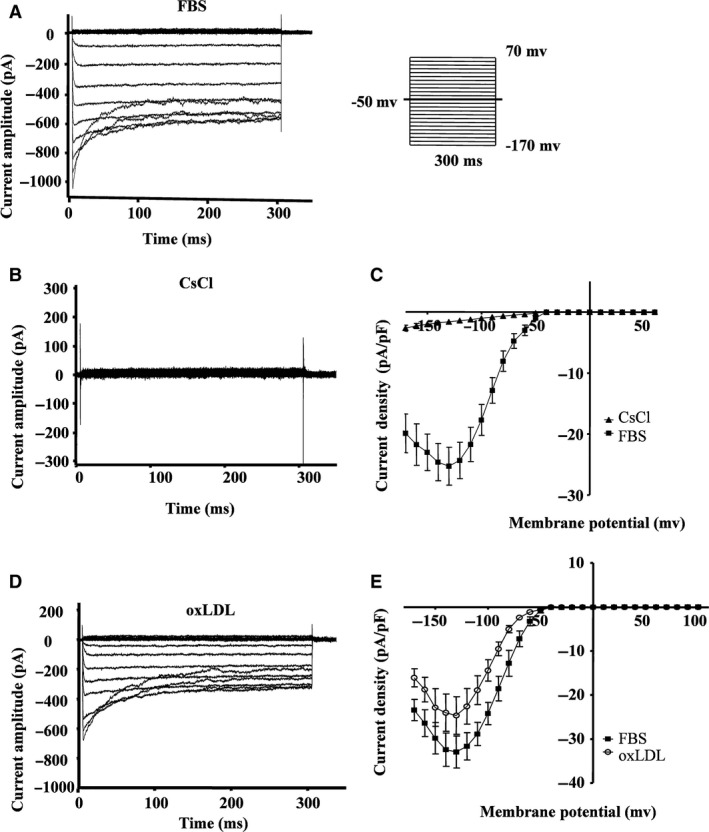
oxLDL induces inward rectifier currents of K^+^ (Kir) channel closure in RAW264.7 cells. Representative traces of RAW264.7 cells current amplitude were documented when cells were exposed to foetal bovine serum (FBS, as control) (**A**) or when the K^+^ was replaced in the bath solution by CsCl to confirm that currents were coming from the potassium channels (**B**) or exposed to oxLDL (30 mg/ml) in the presence of 10% FBS (**D**). Current density (pA/pF) was recorded either when cells were exposed to FBS or after replacement of K^+^ in the bath solution by CsCl (**C**), or exposed to oxLDL (**E**) from a holding potential of −50 mV in 10 mV increments evoked at voltage steps from −170 mV to + 70 mV (mean ± SD from 5 to 10 cells).

RAW264.7 cells were stimulated with voltage steps in the whole‐cell configuration to detect ionic membrane currents. Clamp steps from the holding potential of −50 mV to voltages between −170 and +70 mV elicited the membrane currents as shown in Figure 3A. Hyperpolarizing clamp steps evoked inward rectifier currents and a current density of −24.3 ± 7.3 pA/pF at −120 mV (*n* = 10). These currents exhibited a prominent time‐ and voltage‐dependent inactivation. Depolarizing clamp steps between −40 and +100 mV did not induce any outward currents (Fig. 3C and E). This is a classical Kir current with strong inward rectification, the kinetics of which are similar to those of *Kir2.1*‐encoded K^+^ channel current 34, 35.

To verify the existence of K^+^‐sensitive channels, the effect of the K^+^ channel blocker CsCl (140 mM) was tested. Figure 3B and C shows the current amplitude and current density *versus* voltage, respectively, of the potassium channel when K^+^ in the bath solution was replaced by CsCl. The application of CsCl inhibits the inward rectifier currents, which reduces the inward currents from −24.3 ± 7.3 pA/pF to −1.1 ± 1.2 pA/pF (*n* = 8) at −120 mV (Fig. 3C). Figure 3D and E shows the current amplitude and current density *versus* voltage, respectively, of the potassium channel when cells were exposed to oxLDL (30 μg/ml). The amplitude of the inward current density was reduced from −24.1 ± 9.4 pA/pF to −14.4 ± 6.8 pA/pF (*n* = 9) at −100 mV (Fig. 3E).

### oxLDL‐induced Kir channel repression is TLR‐4‐, ERK‐ and JNK MAPK‐dependent

**Figure 4:**
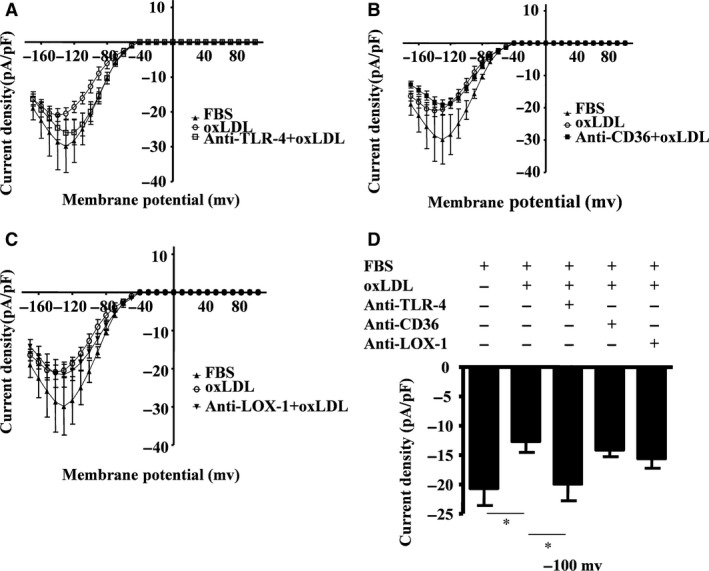
oxLDL‐induced inward rectifier K^+^ channel closure is TLR‐4‐dependent. Current density (pA/pF) in RAW264.7 cells was documented when cells were exposed to foetal bovine serum (FBS, as control) or oxLDL (30 mg/ml) in the presence of 10% FBS, or in some cases pre‐treated with the blocking antibodies of TLR‐4 (8 μg/ml) (**A**), CD36 (8 μg/ml) (**B**) or LOX‐1 (8 μg/ml) (**C**). Histogram showing current density of cells specifically evoked at −100 mV when the cells were pre‐treated with these antibodies (**D**) (mean ± SD from 5 to 10 cells). *P = 0.028, one‐way anova. All data are shown as means ± S.D.

Only the TLR‐4 blocking antibody (Fig. 4A) but not the CD36 or LOX‐1 blocking antibodies (Fig. 4B and C) inhibited oxLDL‐induced potassium inward rectifier currents. The amplitude of the inward current density at −100 mV in the presence of the TLR‐4 blocking antibody increased from −12.7 ± 5.1 pA/pF to −20.0 ± 7.5 pA/pF (*n* = 9) (Fig. 4D). These results indicate that oxLDL‐induced inhibition of inward currents of potassium is TLR‐4‐dependent.

**Figure 5:**
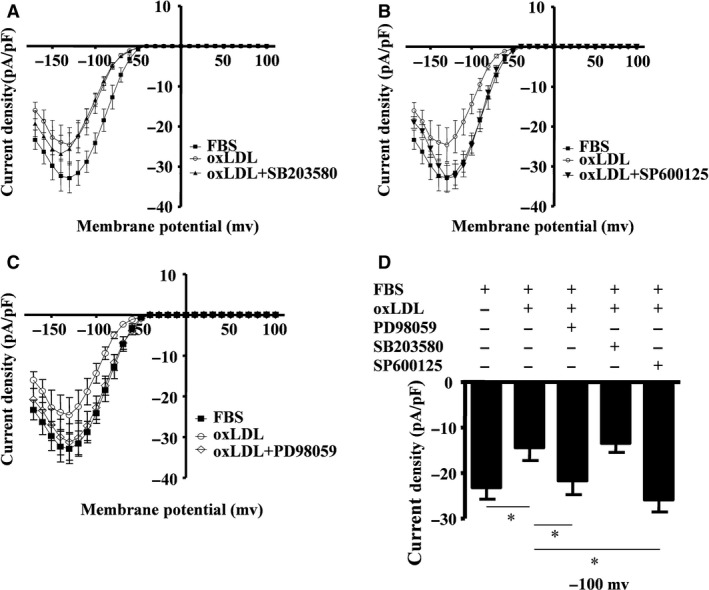
oxLDL‐induced inward rectifier K^+^ channel closure is ERK‐ and JNK MAPK‐dependent. Current density (pA/pF) in RAW264.7 cells was documented when the cells were exposed to foetal bovine serum (FBS, as control) or oxLDL (30 mg/ml) in the presence of 10% FBS, or in some cases pre‐treated with the p38 inhibitor SB203580 (25 μM) (**A**), the JNK inhibitor SP600125 (10 μM) (**B**) or the ERK inhibitor PD98059 (50 μM) (**C**). Histogram showing current density of cells specifically evoked at −100 mV when the cells were pre‐treated with these inhibitors (**D**) (mean ± SD from 5 to 10 cells). *P = 0.012, one‐way anova. All data are shown as means ± S.D.

Treatment with an inhibitor of p38 (SB203580) did not have any effect on inward currents (Fig. 5A), while inhibitors of JNK (SP600125) and ERK (PD98059) (Fig. 5B and C) both increased the inward currents (*P* < 0.05). The amplitude of the inward current density at −100 mV in the presence of the inhibitors of JNK and ERK increased from −14.4 ± 6.8 pA/pF to −24.8 ± 6.0 pA/pF and to −22.8 ± 8.1 pA/pF, respectively (*n* = 9) (Fig. 5D). These results indicated that oxLDL‐induced inhibition of inward rectifier currents of potassium was both ERK‐ and JNK MAPK‐dependent.

### oxLDL‐induced generation of [Ca^2+^]i oscillations in macrophages is ERK‐ and JNK MAPK‐dependent

**Figure 6:**
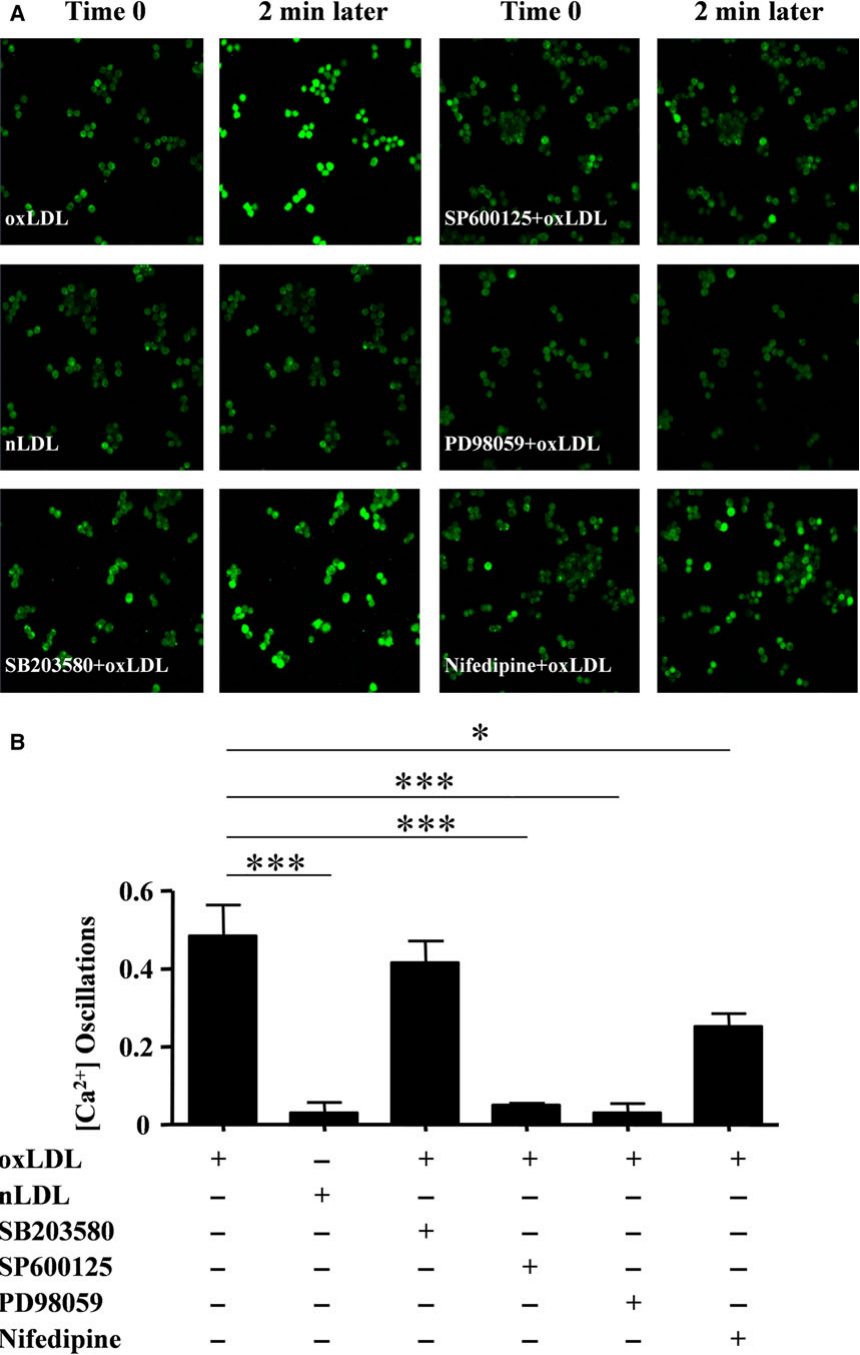
oxLDL induces generation of [Ca^2+^]i oscillations in macrophages that is ERK‐ and JNK MAPK‐dependent. RAW264.7 cells were pre‐treated with the inhibitors PD98059, SP600125, SB203580 and nifedipine, respectively, and thereafter exposed to oxLDL (30 μg/ml) at time 0. Native LDL (nLDL, 30 μg/ml) was used as a control. [Ca^2+^]i oscillations were documented by Fluo‐4‐AM staining. (**A**) Fluorescence image of [Ca^2+^]i oscillations at time 0 and 2 min. later. (**B**) Percentages of [Ca^2+^]i oscillation‐positive cells [mean ± SD (*n* = 3)]. *P = 0.023, ***P = 0.0001, one‐way anova. All data are shown as means ± S.D.

oxLDL has been shown to induce an increase in the intracellular calcium concentration ([Ca^2+^]_i_) in bone marrow‐derived macrophages 36. Here, we tested whether oxLDL influences the calcium influx in RAW264.7 cells. Cells were loaded with the fluorescent calcium dye Fluo‐4 in PBS buffer to visualize the fluctuation of the intracellular Ca^2+^ level in RAW264.7 cells. Cells were pre‐treated with or without inhibitors of ERK, JNK and p38, respectively, for 30 min. and thereafter stimulated with oxLDL. Native LDL (nLDL) was used as a control. As shown in Figure 6A and B, oxLDL induced a high percentage of [Ca^2+^]i oscillations (48.4%) compared to nLDL (3.3%). Moreover, inhibitors of ERK and JNK inhibited the high percentage of oxLDL‐induced [Ca^2+^]i oscillations (3.1% and 5.1%, respectively), whereas the p38 inhibitor had limited effect (41.6%). Taken together, these results indicate that oxLDL induces [Ca^2+^]i oscillations in macrophages and that it is ERK‐ and JNK MAPK‐dependent.

An increase in [Ca^2+^]i can be mediated by an influx of Ca^2+^ from the extracellular environment or from intracellular Ca^2+^ stores. To assess the contribution of extracellular Ca^2+^, RAW264.7 cells were incubated in medium containing nifedipine to block the membrane Ca^2+^ channel. The percentage of Ca^2+^ oscillations in response to oxLDL was reduced by nifedipine (25.2%) to about half of the levels observed in cells incubated without the blocker (Fig. 6B). This indicates that oxLDL‐induced Ca^2+^ oscillations are only partly dependent on the membrane Ca^2+^ channels, which is in accordance with Johnny H. Chen's results showing that not only extracellular Ca^2+^, but also intracellular stores of Ca^2+^ account for the oxLDL‐induced [Ca^2+^]i oscillations 36.

### A recombinant antibody recognizing oxLDL reverses oxLDL‐induced Kir channel repression in a FcγRIIB‐dependent way

**Figure 7:**
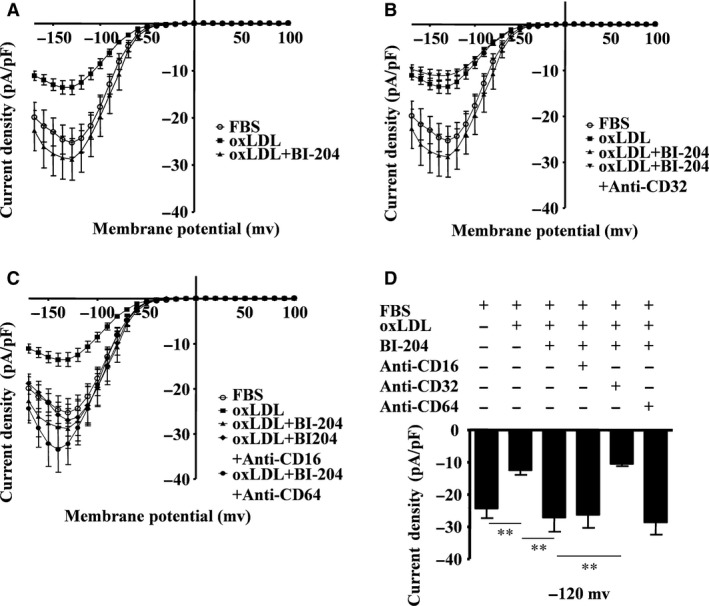
BI‐204 inhibits oxLDL‐induced inward rectifier K^+^ channel closure through FcγRIIB. Current density (pA/pF) in RAW264.7 cells was documented when cells were exposed to foetal bovine serum (FBS, as control) or oxLDL (30 mg/ml) in the presence of 10% FBS, and in some cases pre‐treated with BI‐204 (4 μg/ml) (**A**), the CD32 antibody (3 μg/ml) (**B**), the CD16 antibody (3 μg/ml) or the CD64 antibody (3 μg/ml) (**C**). Histogram showing current density of cells specifically evoked at −120 mV when the cells were pre‐treated with these antibodies (**D**) (mean ± SD from 5 to 10 cells). **P = 0.0073, one‐way anova. All data are shown as means ± S.D.

Treatment with the human recombinant BI‐204 antibody recognizing oxLDL epitopes reversed oxLDL‐induced reduction of inward rectifier currents of K^+^ (Fig. 7A). Moreover, the FcγRIIB (CD32) blocking antibody abolished the BI‐204 effect and reduced the inward rectifier currents (Fig. 7B). However, FcγRI (CD16) and FcγRIII (CD64) blocking antibodies did not have any influence on the BI‐204 effect (Fig. 7C). The amplitude of the inward rectifier current density at −120 mV in the presence of BI‐204 increased from −12.5 ± 4.0 pA/pF to −27.1.0 ± 12.5 pA/pF (*n* = 9), while the CD32 blocking antibody in combination with BI‐204 reduced the current from −27.1.0 ± 12.5 pA/pF to −10.5 ± 1.8 pA/pF (*n* = 9) (Fig. 7D). These results indicated that BI‐204 reversed oxLDL‐induced Kir channel repression in a FcγRIIB‐dependent way.

### oxLDL activates MAPKs, while the antibody recognizing oxLDL (BI‐204) inhibits the ERK MAPK pathway

**Figure 8:**
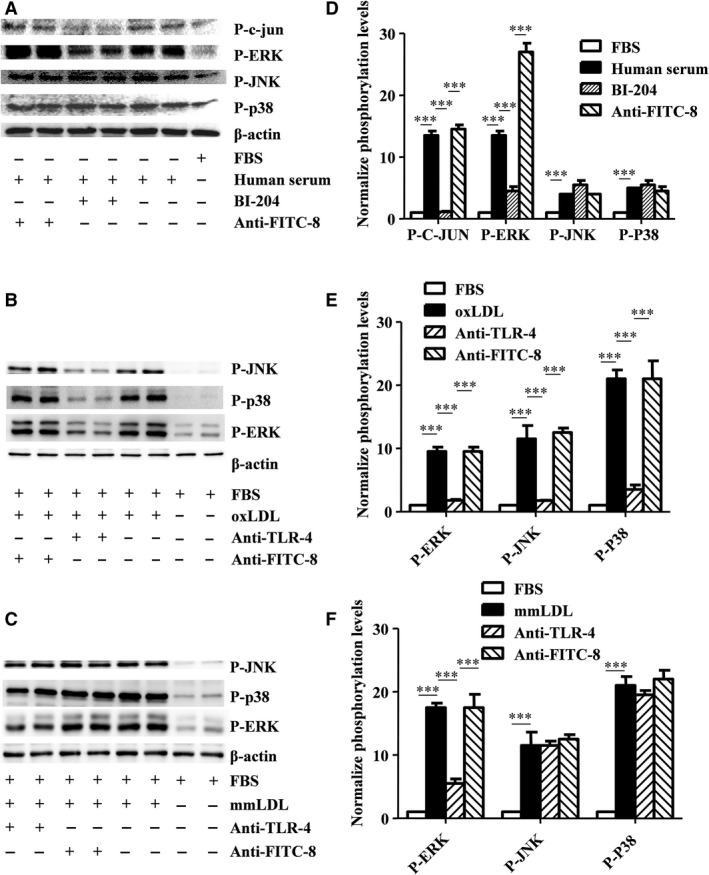
oxLDL activates the ERK pathway through TLR‐4, and the recombinant antibody recognizing oxLDL inhibits human serum‐induced activation of the ERK pathway. CD14^+^ monocytes exposed to either foetal bovine serum (FBS, as control), oxLDL‐containing human serum, Cu^2+^‐oxidized LDL (oxLDL) or Fe^3+^‐oxidized LDL (minimal modified, mmLDL), and in some cases pre‐treated with BI‐204 or the control antibody (FITC‐8) (**A**), or the TLR‐4 blocking antibody or the control antibody (FITC‐8) (**B** and **C**) (*n* = 3). (**D**), (**E**) and (**F**) show normalized quantization of phosphorylation levels for proteins. Gels have been run under the same experimental conditions. ***P = 0.0001, one‐way anova. All data are shown as means ± S.D.

Western blot experiments confirmed that oxLDL‐containing human serum induced p38, ERK and JNK phosphorylation, while the presence of the oxLDL monoclonal antibody (BI‐204) only influenced the ERK activation. The unspecific FITC‐8 antibody was used as a control. To further evaluate the oxLDL monoclonal antibody effect, also phosphorylation of the ERK MAPK substrate, c‐jun, was tested, which confirmed the inhibition of the ERK activity (Fig. 8A). To additionally test whether oxLDL (30 μg/ml for 30 min., which has been verified to be the best stimulation concentration and pre‐treated time in Figure S3A and S3B) induced ERK MAPK phosphorylation, a TLR‐4 blocking antibody was included. We found that the TLR‐4 blocking antibody blocked Cu^2+^‐modified (oxLDL) LDL‐induced activation of MAPKs (Fig. 8B), while the Fe^3+^‐modified LDL (minimal modified LDL, mmLDL) only showed a small effect on the ERK MAPK activation (Fig. 8C).

## Discussion

Toll‐like receptor 4 has been reported responsible for not only oxLDL‐induced cellular signal transduction, but also the uptake of lipids by monocytes/macrophages 12. Our data suggest ERK MAPK plays a key role in both oxLDL‐induced activation and oxLDL mAb inhibition of phagocyte activation. This signal transduction hub is a centre in the signal transduction pathways for the regulation of both ionic channel permeability and eventually MCP‐1 release. That oxLDL mAb inhibits MCP‐1 release reflects its anti‐inflammatory nature and may explain the reason for inhibition of atherosclerosis in the animal experiments.

Cytokine release from phagocytes is known to involve an increased Ca^2+^ concentration in cytoplasm 37. The increment mainly results from a stimulated influx of extracellular Ca^2+^ through VDCCs 17, 38. The degree of membrane hyperpolarization determines the extent of Ca^2+^ influx, while K^+^ influx and efflux maintain the negative potential of the membrane 39. [K^+^]_e_ and [K^+^]_i_ channel activities need to maintain sufficient negative potential to open Ca^2+^ channels and ensuing cytokine release 39. Our results indicate that oxLDL mainly repressed inward rectifier K^+^ channel, to keep the negative charges in macrophages and open Ca^2+^ channels, and we did not detect any outward K^+^ current in RAW264.7 cells, although glyburide, which is an outward K^+^ channel inhibitor, also attenuated oxLDL‐induced MCP‐1 release. It has been reported that calcium stress may activate ERK MAPK 38, and our data further show that ERK MAPK regulates closing and opening of Ca^2+^ and K_ir_ channels and thus regulates MCP‐1 release. Inhibition of oxLDL‐induced MCP‐1 release by other groups of MAPK inhibitors also regulates opening or closing of both ion channels, but oxLDL mAb seems to inhibit oxLDL‐induced ERK MAPK activity only. This is also coincident with the effect of ERK inhibitor on oxLDL‐induced MCP‐1 release; PD98059 is the best dominant inhibitor compared to the other groups of MAPK inhibitors in inhibition of oxLDL‐induced MCP‐1 release. What downstream molecule for FcγRIIB could specifically down‐regulate ERK MAP activity is still unclear.

We previously reported that p38 MAPK is responsible for the signal transduction on activation of macrophage expression of oxLDL‐induced CD36 expression and thus is involved in lipid uptake/metabolism. The p38‐specific inhibitor SB203580 dramatically inhibited oxLDL‐induced foam cell formation from J774 macrophages, while PD98059 played a minor inhibitory effect 40. The differential role of MAPKs in the regulation of lipid metabolism and imbalance of lipid metabolism‐induced inflammatory reaction gives us a more clear understanding of oxLDL‐induced activation of MAPK functions. oxLDL mAb specifically inhibits ERK MAPK pathway but not p38 MAPK, which is coincident with the phenomena in the vaccine and antibody treatment experiment that immune therapies influence only inflammatory effects, but not the lipid metabolisms 7.

**Figure 9:**
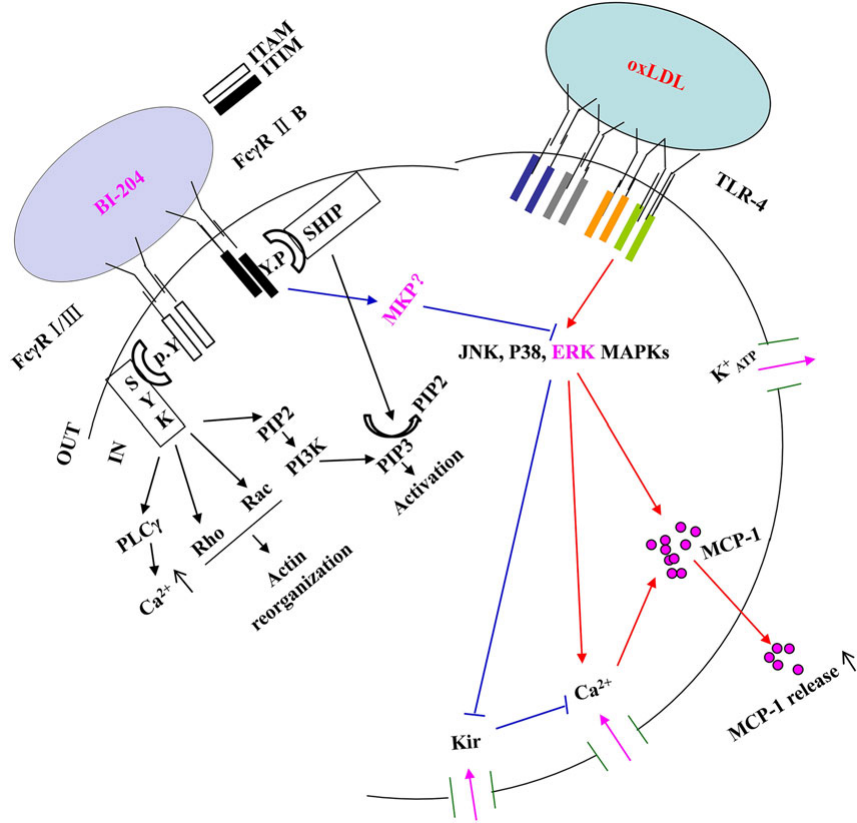
Schematic picture of the oxLDL regulation of MCP‐1 release, and the mechanism of the regulatory effect of oxLDL antibody. oxLDL‐induced MCP‐1 release from monocytes/macrophages is regulated by Ca^2+^ and K^+^ channels, and both of them are TLR‐4‐ and MAPK‐dependent. The oxLDL antibody inhibited oxLDL‐induced Kir channel closure and inhibited the oxLDL‐induced ERK activation through binding to FcγRIIB. Four kinds of Fcγ receptors have been identified: FcγRI, FcγRIIA, FcγRIIB and FcγRIII. FcγRI/III transduces activation signals through its immunoreceptor tyrosine‐based activation motif (ITAM) in cell membranes. Once activated, ITAM recruits spleen tyrosine kinase (SYK) and its downstream targets, such as phospholipase C (PLCγ), GTPase (Rho, Rac) and phosphatidylinositol 3‐kinase (PI3K), to activate macrophages. FcγRIIB transduces inhibitory signals through immunoreceptor tyrosine‐based inhibitory motif (ITIM), which recruits SH2 domain‐containing inositol phosphatase (SHIP), and specifically hydrolyses the PI3K product PIP3 to PIP2. We hypothesize that the oxLDL monoclonal antibody (BI‐204) inhibits oxLDL‐induced macrophage activation through its FcγRIIB by activation of MAPK phosphatase (MKP), which might specifically inactivate the ERK MAPK pathway.

Schiopu *et al*. 7 reported that antibody treatment decreased macrophage content in the plaques and MCP‐1 expression in animal experiments. Although BI‐204 antibody trail (GLACIER) failed in a phase II study in Europe, understanding the immune regulation of atheropathogenesis, especially on antibody‐mediated anti‐inflammatory effects in arterial lesion area, still has its importance both on theoretical understanding of the disease and on future screening and selection of similar or better antibodies than BI‐204, as the immune therapy against atherosclerosis still has a great prospect when we have a more detailed understanding of either molecular mechanism of immune system regulation of lipid metabolism or even more importance of subsequently induced inflammation. Li *et al*. 41 reported that the oxLDL antibody (MLDL1278a) inhibited oxLDL‐induced MCP‐1 release in a full‐length IgG1 format but not in an F(ab′)2 format which lacks the FcγR binding antibody constant region, indicating that antibody effect was by binding to Fcγ receptors and it inhibited p38 MAPK. Our antibody (BI‐204) used in this study was also found to inhibit oxLDL‐induced MCP‐1 release by FcγRIIB (Figure S1). However, BI‐204 specifically inhibited ERK MAPK pathway, so these two antibodies might have different mechanisms of inhibition of macrophage activation (Fig. 9). The detailed molecular mechanism of this difference needs to have further exploration.

Taken together, we have found multiple mechanisms behind oxLDL antibody inhibition of oxLDL‐induced monocyte/macrophage activation and inflammatory chemokine release (Fig. 9). The findings probably partially explained Schiopu's 7 reports that oxLDL antibody treatment had a regression effect on high‐fat diet‐induced atherosclerosis in Apobec‐1^−/−^/LDLR^−/−^ mice, and inhibitory effect on monocyte MCP‐1 release and inflammatory cell infiltration to the plaque area. As many more atherosclerotic antigens are newly found, we speculate that therapeutic effects of antibodies may have more complex mechanisms.

## Authors’ contributions

JS and MZ conceived and designed the study; JS and HZ prepared cells and performed intracellular Ca2+ imaging; JS and XL performed electrophysiological recording; JS and JN performed Western blotting and ELISA; JS, HZ, XL and JN analysed and interpreted the data; JS, MZ, JN and GF wrote the manuscript. All authors have approved the submitted version of the manuscript.

## Competing interests

The authors declare that they have no competing interests.

## Supporting information


**Figure S1** A recombinant antibody recognizing oxLDL inhibits MCP‐1 release through the FcgammaRIIB.
**Figure S2** OxLDL content in human serum and FBS.
**Figure S3** oxLDL induces MAPKs activation dose‐dependently.
**Figure S4** Ion channel and MAPKs inhibitors have no influneces on MCP‐1 release in control cells.
**Figure S5** Freshly prepared oxLDL induces MCP‐1 release through TLR‐4 pathway in monocytes/macrophages.
