# Supplementary material for: oxLDL antibody inhibits MCP‐1 release in monocytes/macrophages by regulating Ca2+/K+ channel flow
Source: J Cell Mol Med. 2016 Dec 20;21(5):929–40. doi: 10.1111/jcmm.13033 (PMC5387129; doi:10.1111/jcmm.13033)

**OxLDL antibody inhibits MCP-1 release in monocytes/macrophages by regulating Ca^2+^/K^+^ channels flow**

Jinyu Su^1^, Hui Zhou^1^, Xianyan Liu^1^, Jan Nilsson^2^, Gunilla Nordin Fredrikson^2^*, Ming Zhao^1^*

^1^Department of Pathophysiology, Key Lab for Shock and Microcirculation Research of Guangdong, Southern Medical University, Guangzhou 510515, P. R. China, ^2^Department of Clinical Sciences, Scania University Hospital, Malmö Lund University, Sweden

Running title: oxLDL regulates MCP-1 release by modulation of Ca^2+^/K^+^ channels

*Corresponding author:

Ming Zhao

Department of Pathophysiology

Southern Medical University, Guangzhou 510515, P.R.China

Tel: +86-20-61648393

Fax: +86-20-61648299

Email: [ming.zhao1966@gmail.com](mailto:ming.zhao1966@gmail.com)

Gunilla Nordin Fredrikson

CRC, Jan Waldenströms gata 35

S-205 02 Malmö, Sweden

Phone: 46 40 391205, Fax: 46 40 391212

Email: Gunilla.Nordin_Fredrikson@med.lu.se

**Supplementary data**

**MATERIALS AND METHODS**

*oxLDL ELISA*

The oxLDL ELISA Kit was applied to test the oxLDL content in human serum and FBS and carried out according to the manufacturer's instructions (Uscn Life Science Inc ). The oxLDL levels in in human serum and FBS was detected by a biotin-labeled antibody and HRP-conjugated streptavidin, and measured at a wavelength of 450 ± 10 nm.

*Western blotting*

Cells were lysed with M-PER Protein Extraction Reagent (Pierce, Rockford, IL) supplemented with protease and phosphatase inhibitor cocktail and protein concentrations of the extracts were measured by bicinchoninic acid (BCA) assay (Pierce). Forty micrograms of the protein was used and loaded per lane, subjected to sodium dodecyl sulfate-polyacrylamide gel electrophoresis (SDS-PAGE), transferred onto nitrocellulose membranes, then blotted as described previously [1]. Detection antibodies used (mentioned above in the section “Materials”) recognized phosphorylation of ERK, JNK and p38. The beta-actin antibody was used as a control.

**RESULTS**

*Recombinant antibody against oxLDL inhibits MCP-1 release through FcgRIIB*

It has been reported that oxLDL mAb inhibits oxLDL-containing human serum -induced monocyte/macrophes release of MCP-1 [2], and enzymatic method made Fab fragment had no such effect (data not shown). To investigate the effect of Fcgamma Receptors, we explored blocking antibody against FcgammaRI (CD64), FcgammaRIIB (CD32), and FcgammaRIII (CD16). Figure S1 shows that, recombinant oxLDL antibody (BI204) significantly induced reduction of oxLDL-containing human serum-induced MCP-1 release from CD14^+^ monocytes, and only CD32 blocking antibody had an inhibitory effect against oxLDL mAb.

*OxLDL induces MAPKs activation dose-dependently.*

OxLDL increased the phosphorylation of ERK, p38, and JNK in a dose-dependent manner (from 10 to 80µg/ml), and MAPK reached the peak of phosphorylaton when monocytes were exposed to 30 to 80 µg/ml oxLDL (Figure 3SA). OxLDL-induced phosphorylation of ERK, p38, and JNK reached its peak after 30 minutes, while after 60 or 90 minutes, it decreased but was still about 10 times higher than the control (Figure 3SB).

*Ion channel and MAPKs inhibitors have no influneces on MCP-1 release in control cells.*

Cells stimulated with FBS were as control, to confirm the inhibitors effect was only on oxLDL-induced MCP-1 release, we exposed these inhibitors respectively to control cells, and Figure S4 shows that Ca^++^ and K^+^ channels inhibitors, nifedipine and glyburide, and SB203580, PD98059 and SP600125 as p38, ERK, and JNK MAPKs inhibitors, all of them had no influences on MCP-1 release in control cells.

**REFERENCES**

[1] **Xu H, An H, Yu Y, et al.** Ras participates in CpG oligodeoxynucleotide signaling through association with toll-like receptor 9 and promotion of interleukin-1 receptor-associated kinase/tumor necrosis factor receptor-associated factor 6 complex formation in macrophages. J Biol Chem 2003;278:36334-40.

[2] **Schiopu A, Frendéus B, Jansson B, et al.** Recombinant Antibodies to an Oxidized Low-Density Lipoprotein Epitope Induce Rapid Regression of Atherosclerosis in Apobec-1−/−/Low-Density Lipoprotein Receptor−/− Mice. J Am Coll Cardiol 2007;50:2313-8.

**FIGURE LEGENDS**

**Figure S1. A recombinant antibody recognizing oxLDL inhibits MCP-1 release through the FcgammaRIIB.** MCP-1 released from CD14^+^ monocytes in culture medium was tested with ELISA. Cells were pretreated with BI-204 and combined with either CD16 (FcgamamRIII), CD32 (FcgamamRIIB), or CD64 (FcgamamRI) blocking antibodies. Thereafter the cells were exposed to fetal bovine serum (FBS), oxLDL containing human serum, or lipoprotein depleted human serum (Lipid-). FITC-8 was used as a control antibody (n=3). **P* = 0.030. ***P* = 0.0065, ****P* = 0.0001, one-way ANOVA. All data were shown as means ± S*.*D.

**Figure S2. OxLDL content in human serum and FBS.** OxLDL content in human serum and FBS was tested by oxLDL ELISA Kit. OxLDL in human serum is about 1296 µg/L, while there is no any oxLDL in FBS. (n=3). ****P* = 0.0001, t-test. Datas were shown as means ± S*.*D.

**Figure S3. oxLDL induces MAPKs activation dose-dependently.** CD14^+^ monocytes were exposed to either fetal bovine serum (FBS, as control), Cu^2+^-oxidized LDL (oxLDL) in different concentration (10, 20, 30, 40, 60, 80μg/ml) in the presence with 10% FBS(A) and for different time (30, 60, 90 min) (B) (n=3). (C) and (D) are normalized quantization of phosphorylation levels for proteins. Gels have been run under the same experimental conditions. ****P* = 0.0001, one-way ANOVA. All data were shown as means ± S*.*D.

**Figure S4. Ion channel and MAPKs inhibitors have no influneces on MCP-1 release in control cells.** MCP-1 release from CD14^+^ monocytes in culture medium was tested with ELISA. Primary CD14^+^ monocytes were treated with or without different concentration of glyburide (1 nM, 10 nM, 100 nM, 1 µM) (A), nifedipine (1 µM, 10 µM and 100 µM) (B) and inhibitors of JNK (SP600125), ERK (PD98059) and p38 (SB203580) MAPKs (C), respectively and then exposed to FBS. OxLDL which in the presence with 10% FBS was as positive control (n=3). ****P* = 0.0001, one-way ANOVA. All data were shown as means ± S*.*D.

**Figure S5. Freshly prepared oxLDL induces MCP-1 release through TLR-4 pathway in monocytes/macrophages.** MCP-1 release from CD14^+^ monocytes in culture medium was tested with ELISA. Primary CD14^+^ monocytes were treated with or without CD36, TLR-4, SR-AI and LOX-1 blocking antibodies, respectively and then exposed to FBS or oxLDL (30 mg/ml) which in the presence with 10% FBS. ****P* = 0.0001, one-way ANOVA. All data were shown as means ± S*.*D.


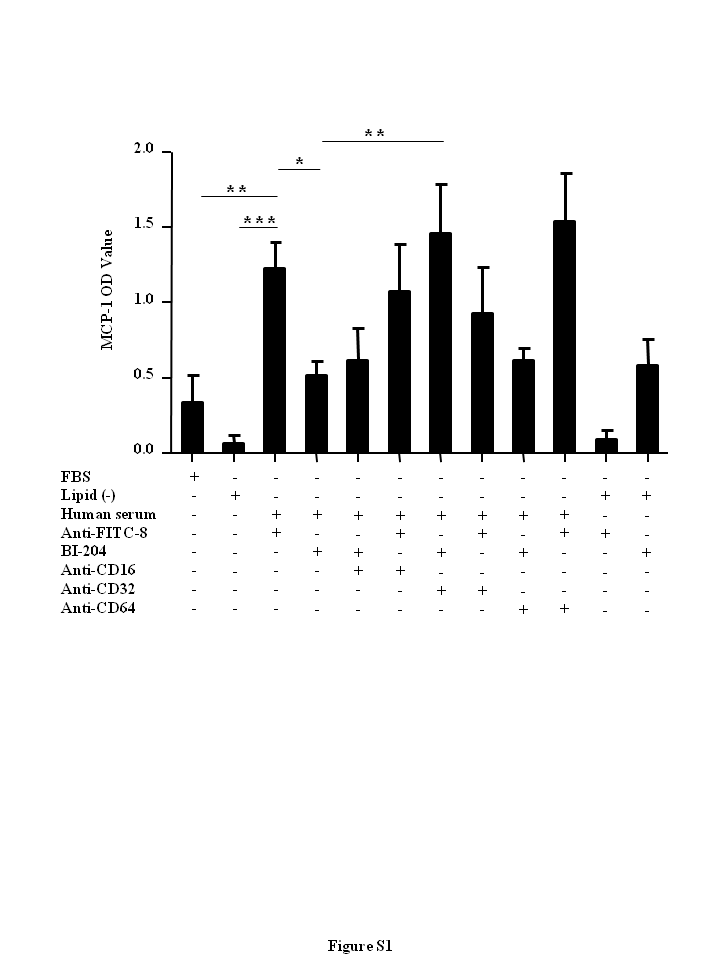

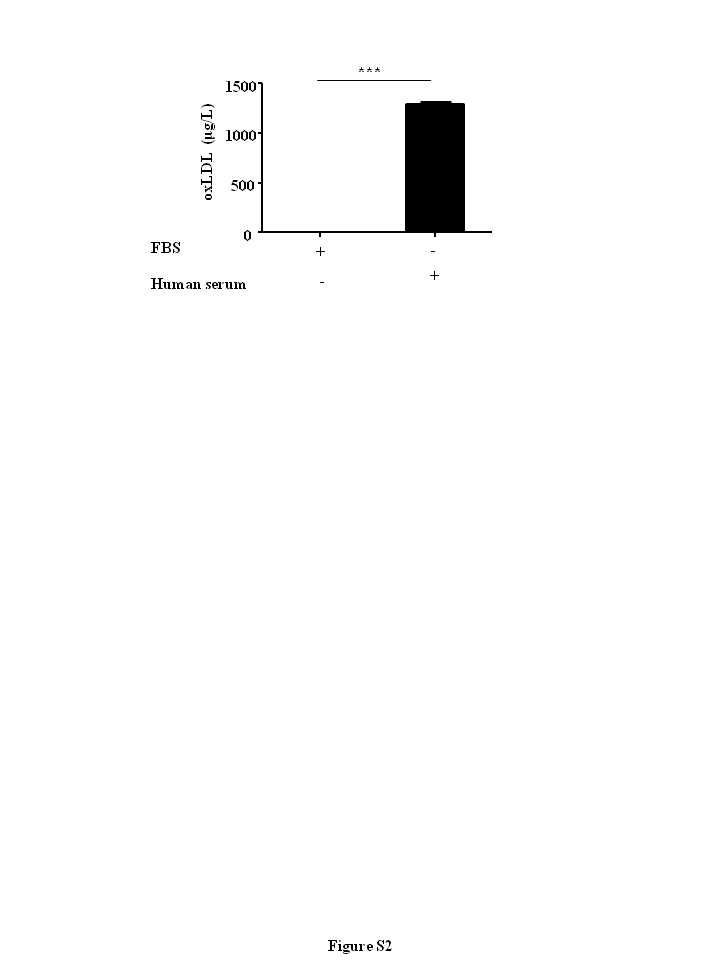

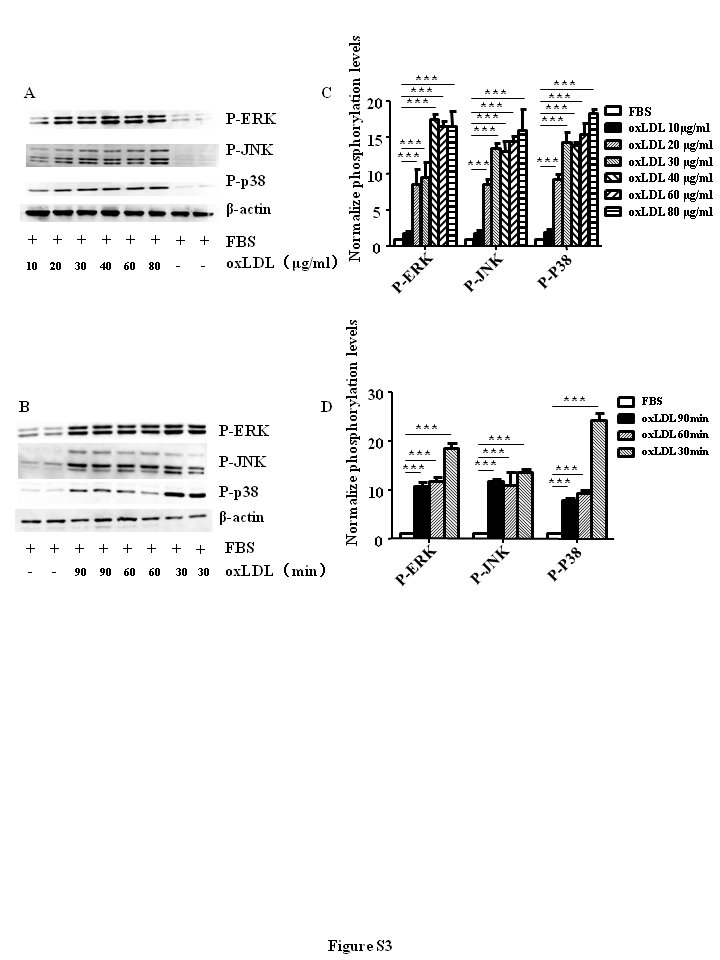

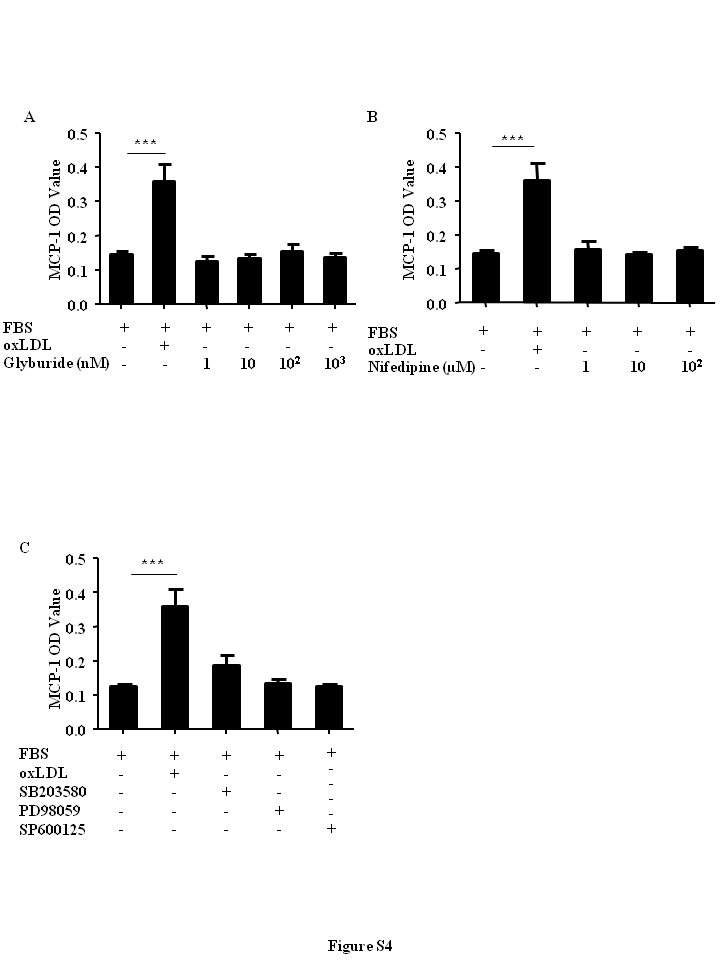

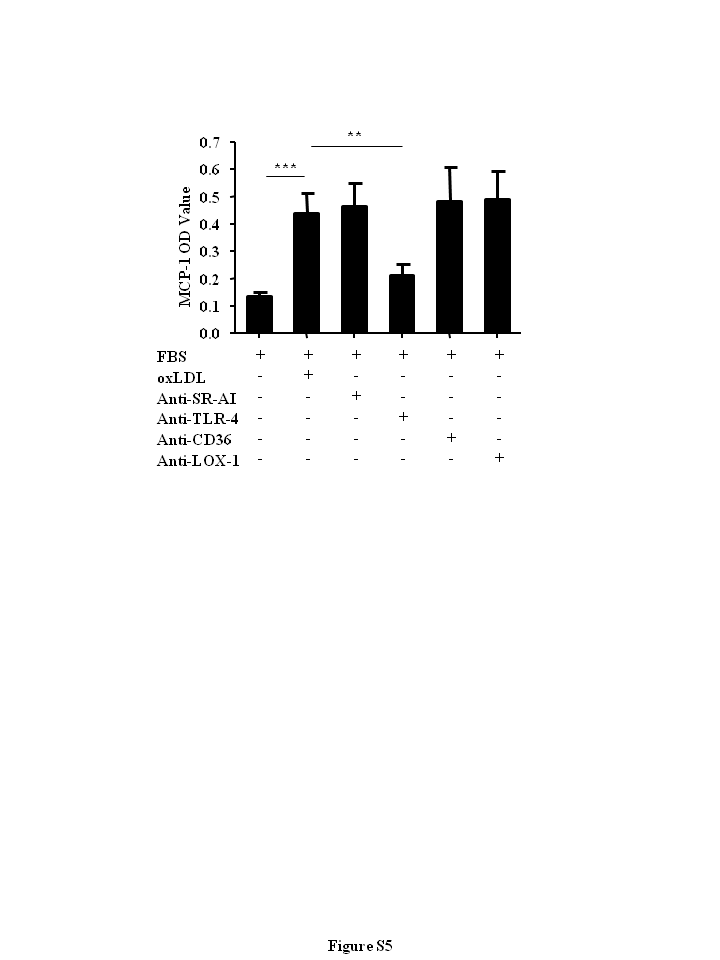

Supplement: Supplementary file 1 — Figure S1 A recombinant antibody recognizing oxLDL inhibits MCP‐1 release through the FcgammaRIIB. Figure S2 OxLDL content in human serum and FBS. Figure S3 oxLDL induces MAPKs activation dose‐dependently. Figure S4 Ion channel and MAPKs inhibitors have no influneces on MCP‐1 release in control cells. Figure S5 Freshly prepared oxLDL induces MCP‐1 release through TLR‐4 pathway in monocytes/macrophages. [file JCMM-21-929-s001.doc]
